# Laparoscopic Local Resection via Duodenotomy for Cystic Heterotopic Pancreas Involving the Gastroduodenal Junction: A Case Report

**DOI:** 10.70352/scrj.cr.26-0191

**Published:** 2026-06-11

**Authors:** Atsushi Gakuhara, Ryugo Teranishi, Yosuke Yamahira, Nozomi Mikami, Kurumi Tsuchihashi, Soichiro Minami, Wataru Fujii, Chikato Koga, Naotsugu Haraguchi, Tomoko Wakasa, Yutaka Kimura

**Affiliations:** 1 Department of Gastroenterological Surgery, Kindai University Nara Hospital, Ikoma, Nara, Japan; 2 Department of Pathology, Kindai University Nara Hospital, Ikoma, Nara, Japan

**Keywords:** heterotopic pancreas, gastric outlet obstruction, retention cyst, duodenotomy, laparoscopic surgery

## Abstract

**INTRODUCTION:**

Heterotopic pancreas is an uncommon condition characterized by the pancreatic tissue lacking anatomical continuity with the main pancreas. Cystic changes are rare, and lesions involving the gastroduodenal junction present diagnostic and technical challenges because the exact site of origin may be difficult to determine.

**CASE PRESENTATION:**

A 53-year-old man presented with epigastric fullness. Endoscopy and imaging revealed food retention in the stomach and a 4.5-cm cystic submucosal lesion in the duodenal bulb. Endoscopic ultrasonography revealed a predominantly anechoic cystic lesion. However, the layer of origin could not be determined. CT and MRI revealed no solid components or continuity with the pancreas. Although no definitive malignant features were observed, duplication cysts or mesenchymal tumors could not be excluded. Laparoscopic surgery was performed for both diagnostic and therapeutic purposes. Intraoperatively, a lesion was found near the pyloric ring that protruded into the duodenum; however, this reduction was unsuccessful. A longitudinal duodenotomy was performed to deliver the tumor into the abdominal cavity, and the lesion was resected, including its base. The defect was closed transversely using a 2-layer Albert–Lembert closure, and intraoperative endoscopy confirmed luminal patency. Histopathological examination revealed a Heinrich type II heterotopic pancreas with retention cyst formation. Pathological mapping demonstrated that the ductal opening was identified on the duodenal mucosal side, and no heterotopic pancreatic tissue was exposed at the resection margin. No dysplasia, pancreatic intraepithelial neoplasia, or malignant transformation was observed. Both the gastric and duodenal mucosa were identified on the lesion surface, indicating involvement of the gastroduodenal junction. The postoperative course was uneventful.

**CONCLUSIONS:**

A cystic heterotopic pancreas involving the gastroduodenal junction may preclude precise preoperative identification of its origin. When reduction is not feasible, duodenotomy allows direct assessment of the lesion base and facilitates appropriate resection while avoiding distal gastrectomy and maintaining gastric outlet patency.

## Abbreviations

CA19-9
: carbohydrate antigen 19-9
CEA
: carcinoembryonic antigen
EUS
: endoscopic ultrasonography
EUS-FNA
: endoscopic ultrasound-guided fine-needle aspiration
LECS
: laparoscopic and endoscopic cooperative surgery

## INTRODUCTION

Heterotopic pancreas is defined as pancreatic tissue lacking anatomical and vascular continuity with the main pancreas.^1)^ It is a relatively uncommon condition, reported in 1%–2% of autopsy cases,^2)^ and most frequently occurs in the stomach and duodenum.^3)^ Although most patients are asymptomatic, symptoms may arise due to inflammation, bleeding, obstruction, or, rarely, malignant transformation.

The heterotopic pancreas is histologically classified according to the system originally proposed by von Heinrich^4)^ and later modified by Gaspar-Fuentes et al.^5)^

Cystic changes in the heterotopic pancreas are rare and are thought to result from ductal obstruction, leading to retention cyst formation.^6)^ When a cystic lesion is identified, differentiating it from duplication cysts, Brunner’s gland lesions, lymphangiomas, and mesenchymal tumors is often difficult based on imaging findings alone.^7,8)^

The pyloric ring represents the anatomical border between the stomach and the duodenum. It may be difficult to determine the origin of lesions in this region. In addition, the extent of resection must be carefully considered because postoperative passage may be affected. We report a case of cystic heterotopic pancreas involving the gastroduodenal junction with extension into the duodenum, in which duodenotomy was required because the lesion could not be reduced.

## CASE PRESENTATION

A 53-year-old man presented with epigastric fullness. Upper gastrointestinal endoscopy and contrast-enhanced CT performed at another hospital revealed food retention in the stomach and a submucosal tumor–like lesion in the duodenal bulb. The patient was referred to our institution for evaluation and management.

**Fig. 1:**
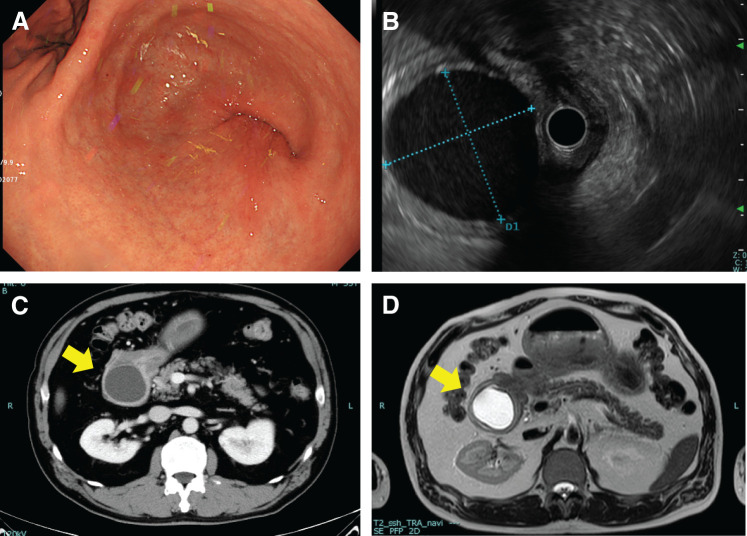
Preoperative imaging and endoscopic findings. (**A**) Upper gastrointestinal endoscopy showing a submucosal tumor-like protrusion extending into the duodenal lumen near the pyloric ring. (**B**) EUS demonstrated a 48 × 42-mm cystic lesion, predominantly anechoic with partially hyperechoic floating components; the layer of origin could not be clearly identified. (**C**) Contrast-enhanced CT showed a well-defined cystic lesion in the duodenal bulb measuring approximately 4.5 cm, without an obvious solid component and without continuity with the pancreas. The yellow arrow indicates the cystic mass. (**D**) MRI showed a cystic lesion with high signal intensity on T2-weighted images, consistent with a fluid-containing cystic mass in the duodenal bulb (yellow arrow). EUS, endoscopic ultrasonography

Endoscopy revealed retained gastric contents and mild narrowing of the pylorus, although the scope could be advanced into the duodenum. A submucosal tumor–like protrusion was observed on the anterior wall of the duodenal bulb. The lesion appeared to extend from the region near the pyloric ring into the duodenum; however, it was not possible to determine whether it originated in the stomach or the duodenum (**Fig. 1A**).

EUS revealed a 48 × 42-mm cystic lesion with homogeneous anechoic contents and partially hyperechoic floating material. The lesion appeared to arise within the gastrointestinal wall; however, the layer of origin could not be clearly identified (**Fig. 1B**). Contrast-enhanced CT revealed a 4.5-cm cystic mass in the duodenal bulb without solid components or continuity with the pancreas (**Fig. 1C**). MRI revealed high signal intensity on T2-weighted images. No dilatation of the bile or pancreatic ducts was observed (**Fig. 1D**). EUS-FNA was not performed because the lesion was predominantly cystic and lacked an apparent solid component that could serve as a safe and diagnostically useful target. In addition, potential risks associated with the puncture of a cystic lesion, such as cyst rupture, leakage, or infection, were considered. Cyst fluid tumor markers, including CEA and carbohydrate antigen 19-9 (CA19-9), were not measured.

Although no definitive malignant features were identified, duplication cysts and mesenchymal tumors could not be excluded. A duplication cyst was considered because the lesion appeared to arise within the gastrointestinal wall and had cystic characteristics. A mesenchymal tumor with cystic degeneration also could not be excluded because the layer of origin could not be clearly determined by EUS. As the patient had obstructive symptoms and a definitive diagnosis could not be established, surgical resection was planned.

**Fig. 2:**
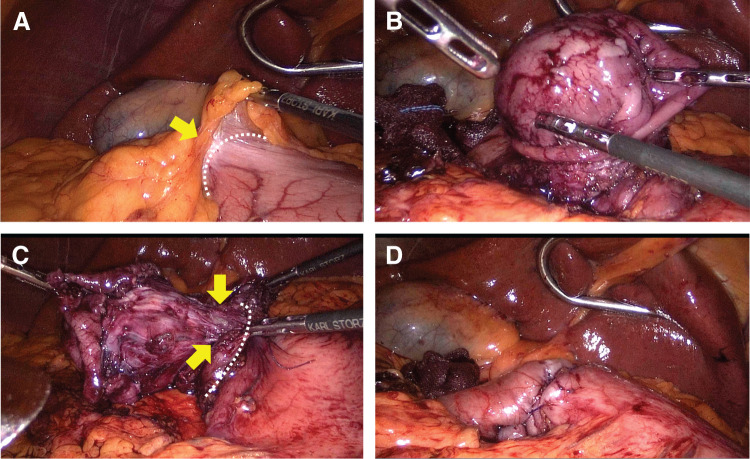
Intraoperative findings. (**A**) Laparoscopic view demonstrating adhesion of the greater omentum to the anterior wall of the antrum near the pyloric ring. The white dotted line indicates the pyloric ring, and the yellow arrow indicates the lesion site. (**B**) After longitudinal duodenotomy on the anterior wall of the duodenal bulb, the tumor was delivered into the abdominal cavity. (**C**) The base of the lesion was located near the pyloric ring after careful dissection. The white dotted line indicates the pyloric ring, and the yellow arrows indicate the base of the lesion. (**D**) Appearance after transverse 2-layer Albert–Lembert closure of the duodenal wall.

Laparoscopic surgery was performed with the patient in the supine position. Intraoperatively, adhesions of the greater omentum were observed near the anterior wall of the pyloric ring (**Fig. 2A**). Intraoperative endoscopy revealed that the mass protruded into the duodenal lumen from the region near the pyloric ring. Reduction into the gastric lumen was attempted, but was unsuccessful. Laparoscopic and endoscopic cooperative surgery (LECS) was considered as a possible local resection strategy. However, because the lesion could not be reduced into the gastric lumen and its base could not be adequately visualized endoscopically, LECS or simple intraluminal resection was not considered feasible. Therefore, a longitudinal incision was made on the anterior wall of the duodenal bulb, and the tumor was delivered into the abdominal cavity (**Fig. 2B**). The base of the lesion was located near the pyloric ring and exhibited inflammatory changes (**Fig. 2C**). The lesion, including the base, was resected.

The duodenal defect was closed transversely using a 2-layer Albert–Lembert closure, in a manner conceptually similar to a Heineke–Mikulicz-type closure, to avoid luminal narrowing near the pyloric ring (**Fig. 2D**). Intraoperative endoscopy confirmed an adequate luminal patency without stenosis. A drain was placed, and the procedure was completed. The operative time was 4 h 39 min, and blood loss was 76 mL.

**Fig. 3:**
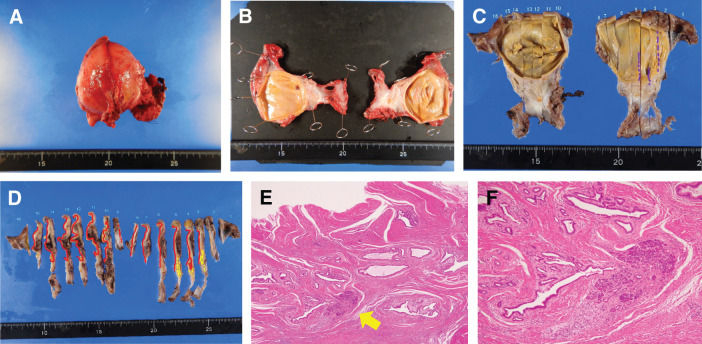
Macroscopic and microscopic findings. (**A**) Resected specimen showing a cystic lesion measuring approximately 4.5 cm filled with fluid and without an apparent solid component. (**B**) Cut surface of the specimen demonstrating a cystic cavity containing fluid. (**C**) Cut surface of the cystic lesion. The purple dotted lines indicate the areas where heterotopic pancreatic tissue was identified. (**D**) Macroscopic pathological mapping of serial sections. The red lines indicate the cyst wall, and the yellow dots indicate the distribution of heterotopic pancreatic tissue. The ductal opening was identified on the duodenal mucosal side. No heterotopic pancreatic tissue was exposed at the resection margin. (**E**) Hematoxylin and eosin staining (×40) showing pancreatic acinar cells and ductal structures located within the muscularis propria (yellow arrow). Islets of Langerhans were not identified. (**F**) Higher magnification view (hematoxylin and eosin, ×100) showing pancreatic acini and ducts within the muscularis propria.

The resected specimen measured approximately 4.5 cm and contained a cystic lesion filled with fluid (**Fig. 3A** and **3B**). Pathological mapping demonstrated that the heterotopic pancreatic tissue was distributed around the cyst wall and the base of the lesion, and the ductal opening was identified on the duodenal mucosal side (**Fig. 3C** and **3D**). A histopathological examination revealed a cyst in the muscularis propria of the gastrointestinal wall. Both gastric and duodenal mucosa were identified on the surface of the lesion, but the exact site of origin could not be determined. Pancreatic acini and ductal structures were identified, whereas islets of Langerhans were not confirmed, consistent with a Heinrich type II heterotopic pancreas with retention cyst formation^4)^ (**Fig. 3E** and **3F**). No heterotopic pancreatic tissue was exposed at the resection margin, and complete excision was considered to have been achieved. No dysplasia, pancreatic intraepithelial neoplasia, or malignant transformation was observed.

The postoperative course was uneventful. The patient was discharged on POD 8. Follow-up endoscopy revealed no suture-site stenosis. The patient remained asymptomatic without evidence of recurrence for 2 years after surgery.

## DISCUSSION

This case highlights the diagnostic challenges and surgical decision-making required for a cystic heterotopic pancreas located near the pylorus. In large clinicopathologic series, most heterotopic pancreas lesions are asymptomatic and incidentally detected, whereas only a minority become symptomatic enough to require intervention.^2,3)^ Symptoms are more likely to occur when lesions enlarge, become inflamed, or mechanically narrow the lumen. Although heterotopic pancreas most frequently occurs in the stomach and duodenum, cystic transformation itself has no clearly established preferential site. Rather, lesions near the pylorus may become symptomatic more readily because even moderate enlargement can cause luminal narrowing or gastric outlet obstruction.

Cystic changes in the heterotopic pancreas are uncommon and are generally attributed to ductal obstruction, leading to retention cyst formation.^6)^ When cystic degeneration or pseudocyst formation develops within heterotopic pancreatic tissue, progressive enlargement may result in luminal narrowing or gastric outlet obstruction, particularly in anatomically confined areas such as the pylorus.^9,10)^ As summarized in **Table 1**,^11–16)^ most reported cases of cystic heterotopic pancreas presenting with obstructive symptoms occur in the gastric antrum or pyloric region. Preoperative differentiation from duplication cysts, Brunner’s gland lesions, and mesenchymal tumors is often difficult because imaging findings are nonspecific.^7,8)^ In the present case, the lesion was predominantly cystic and lacked continuity with the pancreas. These findings did not strongly suggest invasive malignancy; however, they were not specific enough to establish a definitive diagnosis. The presence of ductal structures and a ductal opening on the duodenal mucosal side supports the possibility that impaired ductal drainage contributed to retention cyst formation. In some cases, obstructive lesions caused by heterotopic pancreas have been misinterpreted as malignant tumors, resulting in more extensive resections than necessary.^17)^ This underscores the importance of careful intraoperative assessment and individualized surgical decision-making.

**Table 1:** Reported cases of cystic heterotopic pancreas near the pylorus or duodenal bulb

Author (year)	Age	Sex	Location	Symptoms	Size (mm)	Heinrich’s classification	Treatment	Outcome
Jung et al. (2011)^11)^	21	F	Gastric antrum	Abdominal pain, vomiting	75	III	Open subtotal gastrectomy	Symptom-free (8 mo)
Jin et al. (2017)^12)^	40	M	Gastric antrum	Abdominal pain, nausea, vomiting	25	Not described	EUS-guided drainage with stent placement	Symptom-free (4 yr)
Iwahashi et al. (2019)^13)^	40	M	Gastric antrum	Abdominal pain, vomiting	60	III	Open distal gastrectomy	Symptom-free (12 mo)
Kawaguchi et al. (2023)^14)^	75	M	Duodenal bulb	Asymptomatic	60	II	Open distal gastrectomy	Uneventful
Mirzaie et al. (2023)^15)^	43	M	Gastric antrum	Abdominal pain, vomiting	40	II	Laparoscopic distal gastrectomy	Uneventful
Fukuda et al. (2025)^16)^	46	M	Gastric antrum	Abdominal pain, vomiting	40	II	Laparoscopic distal gastrectomy	Symptom-free (7 mo)
Present case	53	M	Gastroduodenal junction	Epigastric fullness, gastric stasis	48	II	Laparoscopic local resection with duodenotomy	Symptom-free (2 yr)

EUS, endoscopic ultrasonography; F, female; M, male; mo, months; yr, years

Several treatment strategies have been developed to treat this condition. In many of the cases listed in **Table 1**, distal gastrectomy was selected, especially when malignancy could not be excluded or when the lesion was large and symptomatic. While effective, these procedures sacrifice normal gastric tissue and may affect pyloric function. In contrast, endoscopic US-guided transmural drainage with stent placement has been reported in selected cases of pseudocysts associated with heterotopic pancreas.^12)^ This minimally invasive approach may relieve symptoms in cases where cystic changes are secondary to inflammation and malignancy is unlikely. However, unlike pseudocysts, retention cysts result from ductal obstruction within the heterotopic tissue itself and structural abnormalities persist unless the tissue is removed.^6)^ EUS-guided drainage does not address the underlying heterotopic pancreatic tissue and may carry a risk of recurrence or incomplete resolution, particularly when the lesion involves the muscularis propria or has a broad base near the pyloric ring.^12)^

In this case, several factors influenced the choice of surgical management. First, the lesion was located near the gastroduodenal junction, and its precise origin could not be determined preoperatively. Although the ductal opening on the duodenal mucosal side suggested a close relationship with the duodenal mucosa, both gastric and duodenal mucosa were identified on the lesion surface. Therefore, the lesion was considered to involve the gastroduodenal junction, and its exact anatomical origin could not be definitively determined. Second, reduction into the gastric lumen was not feasible, making simple intraluminal excision difficult. Third, the base of the lesion was situated close to the pyloric ring, where inadequate resection might have resulted in recurrence, whereas excessive resection could have impaired gastric emptying. At the gastroduodenal junction, identifying the exact origin of the lesion is clinically important because it directly influences the surgical strategy. If the lesion had clearly originated from the gastric antrum and could have been reduced into the stomach, intragastric or LECS-based local resection might have been considered. Conversely, if malignancy had been strongly suspected, if the base had been broad and unresectable locally, or if safe preservation of the pyloric ring had not been possible, distal gastrectomy would have been considered. Duodenotomy allowed direct visualization of the lesion base and facilitated accurate determination of the resection margin while avoiding distal gastrectomy. The absence of postoperative stenosis and the favorable 2-year clinical course support the adequacy of this approach.

Compared with previously reported cases summarized in **Table 1**, the present case demonstrates that local resection with duodenotomy can be considered as an alternative to distal gastrectomy in carefully selected patients. Accurate intraoperative assessments of reducibility and the lesion base are essential for determining the optimal extent of resection. Although malignant transformation of heterotopic pancreas is rare, occult dysplastic changes or pancreatic intraepithelial neoplasia have been reported.^18,19)^ Therefore, a complete pathological assessment is important, particularly for large or symptomatic lesions. In the present case, no dysplasia, pancreatic intraepithelial neoplasia, or malignant transformation was identified, supporting the adequacy of local resection.

This report has limitations as it describes a single case. First, EUS-FNA and cyst fluid analysis, including CEA and CA19-9 measurements, were not performed because the lesion was predominantly cystic and lacked an apparent solid target. Second, additional retroflexed endoscopic views from the duodenal bulb were not available because retroflexion was not feasible due to luminal narrowing caused by the lesion. Third, although the follow-up period was limited to 2 years, no recurrence has been observed to date.

## CONCLUSIONS

A cystic heterotopic pancreas involving the gastroduodenal junction presents diagnostic and technical challenges. When reduction is not feasible, duodenotomy may allow direct assessment of the lesion base and facilitate appropriate resection while avoiding distal gastrectomy and maintaining gastric outlet patency. This case suggests that local resection with duodenotomy can be a practical surgical option for selected junctional lesions.

## DECLARATIONS

### Availability of data and materials

All data generated or analyzed during this study are included in this published article.
